# “There’s So Much More Support We Could Have Provided”: Child Life Specialists’ Stories of the Challenges Working in Adult Oncology

**DOI:** 10.1177/10497323231215950

**Published:** 2023-11-30

**Authors:** Shipra Taneja, Meredith Vanstone, David L. Lysecki, Heather McKean, Daryl Bainbridge, Jonathan Sussman, Monica Molinaro

**Affiliations:** 1 Department of Family Medicine, 3710 McMaster University, Hamilton, ON, Canada; 2 Department of Pediatrics, 3710 McMaster University, Hamilton, ON, Canada; 3 Hamilton Health Sciences, 103398 McMaster Children’s Hospital, Hamilton, ON, Canada; 4 Department of Oncology, 3710 McMaster University, Hamilton, ON, Canada; 5 Institute of Health Sciences Education, McGill University, Montreal, QC, Canada

**Keywords:** oncology, child life specialist, palliative care, narrative, challenges, stress, systemic barriers, death

## Abstract

A cancer diagnosis in patients who are parents of minor children is uniquely stressful for both parents and children. Children need developmentally appropriate information and support to help reduce their fears and worries. Child life specialists (CLSs) are health professionals who work in pediatric environments to support children and families with the stress and uncertainty of illnesses. Increasingly, CLSs have been called upon to support children of patients in adult clinical environments. Our objective was to elucidate CLS caregiving narratives related to working with children of adult cancer patients. We used narrative inquiry to interview four CLSs working in adult oncology. Canadian CLSs who have experience providing care for children and families affected by parental cancer were recruited via convenience sampling. We used narrative analysis methods that included multiple close reads of the data, generating narrative themes, and noting conflicts or tensions in the data. CLSs’ caregiving stories often highlighted the complexities of working in an adult oncology environment. Their narratives included challenges in providing optimal care to the children, including family-level barriers (such as parental wishes to withhold information from their children) and systemic barriers (such as late referrals and limited options for bereavement support). CLS participants identified several challenges of working with families in adult oncology. The CLSs highlighted a desire for additional institutional support for children of adult oncology patients and for themselves working in these environments in order to achieve what they believed to be optimal care.

## Introduction

While most research on experiences of cancer focuses on the person diagnosed (the “patient”), a cancer diagnosis can affect the entire family unit (Inhestern et al., 2021; Semple & McCaughan, 2013). Fourteen to twenty-five percent of patients who receive cancer diagnoses are parents of young children or adolescents (Inhestern et al., 2021; Rashi et al., 2015). These patients must balance their own health needs with their parental responsibilities (Davey et al., 2012; Weaver et al., 2010). Their children need to adjust their daily routine while attempting to cope with the stress and uncertainty of diagnosis (Ellis et al., 2017; Inhestern et al., 2021; Morris et al., 2016; Tafjord, 2021). Children who are affected by parental cancer are at increased risk of psychological distress and psychosocial difficulties, such as separation anxiety, depression, reduced self-esteem, post-traumatic stress disorder, and internalizing and externalizing problems (Alexander et al., 2019; Levesque & Maybery, 2012; Morris et al., 2016, 2018). They may also be asked to take on extra roles and responsibilities at home, to support their ill parent (Huizinga et al., 2011; Kennedy & Lloyd-Williams, 2009a).

When a parent is diagnosed with cancer, the oncology team primarily and understandably focuses on the patient’s care. Most clinical teams are not resourced to simultaneously support the informational and emotional needs of the patient’s family members, particularly if these family members include minor children (Alexander et al., 2019). Knowing that children affected by parental cancer are at increased risk of developing emotional and psychological sequelae, previous research has identified that providing children with developmentally appropriate information and support about their family members’ cancer can reduce their fears and worries (Ellis et al., 2017; Phillips & Prezio, 2017). However, oncology care providers may not be prepared due to time constraints and a lack of expertise in pediatric psychosocial needs (Hernandez et al., 2023; Niemelä et al., 2010). Child life specialists (CLSs), however, are health professionals specifically trained to support children, youth, and their families to cope with the uncertainty and stress of illness, injury, trauma, treatment, and grief and bereavement (Humphreys & LeBlanc, 2016; Association of Child Life Professionals, 2022). In the context of adult oncology, child life specialists adopt a family-centered care approach (McGee, 2003; Shields et al., 2007). Once the family is referred to the child life specialist by the oncology team, child life specialists collaborate closely with the adult “patient” and other family members to support the well-being of the child(ren). This approach helps to effectively support the family by making sure the family’s emotional, psychological, and practical needs are taken into consideration. To foster a patient- and family-centered care approach, the child life specialist follows several core principles (Lookabaugh & Ballard, 2018). These principles include actively listening and respecting both the child(ren) and family member, customizing interventions to cater to the unique needs of each child and family, sharing information honestly, offering both formal and informal support, and engaging in a collaborative relationship with families and patients across all levels of healthcare (Lookabaugh & Ballard, 2018). Child life specialists use an array of play interventions to build rapport and support the psychosocial needs of children and adolescents (Humphreys & LeBlanc, 2016; Professionals, 2022). Using these interventions, child life specialists have the tools to adequately support children impacted by parental cancer, whether the child’s experience of parental cancer concerns a past diagnosis, ongoing treatment, or terminal illness.

Most child life specialists provide services in pediatric hospital settings (Bruce & McCue, 2018). Occasionally, child life specialists find themselves providing advice to adult healthcare providers who request consults catalyzed by a child visiting a terminally ill adult patient (Bruce & McCue, 2018). Over the last decade, a number of pilot programs have commenced, identifying the value of incorporating child life specialists to support minor children and families of adults receiving critical, oncological, or palliative care (Bruce & McCue, 2018; Lysecki et al., 2021a, 2021b; Piazza et al., 2021; Sutter & Reid, 2012). Understanding the experience of these child life specialists is important, given that they perform highly emotional and potentially traumatic work which can affect their personal and professional well-being (Fisackerly et al., 2016; Munn et al., 1996; Shuck et al., 2013). For example, child life specialists have described experiences of emotional exhaustion, job stress, frequent turnover, and compassion fatigue stemming from the care they provide to children and their families and the associated working conditions (Fisackerly et al., 2016; Shuck et al., 2013). The limited literature which exists in this area focuses on the experience of child life specialists working in general pediatric settings (Fisackerly et al., 2016; Shuck et al., 2013), while the experiences of child life specialists within the unique care context of adult oncology have not been examined. Research on other professionals working in adult oncology has documented a high likelihood of experiencing moral distress or compassion fatigue (Atli Özbaş et al., 2021; Guan et al., 2021; Laor-Maayany et al., 2020; Simon et al., 2005). To better understand how child life specialists experience work in this setting, we sought to elucidate child life specialists’ caregiving narratives of working in adult oncology.

## Methods

### Study Design and Population

We employed narrative inquiry to elucidate child life specialists’ caregiving stories of working in adult oncology. Narrative inquiry is an umbrella term that encompasses a multitude of research approaches, methods, and assumptions underlaid by a particular interest in stories and storytelling. While narratives can include a variety of sources, including spoken words, written documents, images, or videos, in the context of qualitative research, narrative inquiry can involve collecting stories as data for the purposes of analysis (Emden, 1998; Riessman, 2008). In doing so, both *what* was said in the participants’ stories and *how* the stories were told are potential elements for analysis (Riessman, 2008). This narrative inquiry study was unlaid by a constructivist epistemological position, acknowledging that we create understanding and generate meaning through our interactions (Crotty, 1998).

Understanding that narrative inquiry analyzes the stories of individuals with rich lived experiences, we sought to recruit child life specialists who have experience working in adult oncology and have provided care for children and families affected by parental cancer. We received ethics approval from the Hamilton Integrated Research Ethics Board (Project #7742). We used a combination of convenience and purposive sampling for recruitment. Specifically, recruitment was purposive in that our participants had to be licensed and practicing child life specialists in the province of Ontario; working (currently or within the past 2 years) in adult oncology; able to read, write, and communicate in English; and, able to provide informed consent. Sampling was convenient in that one of the members of the study team contacted child life specialists within their network who may be interested in participating. Potential participants were sent a brief email with information on the study, including the contact information of the lead authors (ST and MM) if they were interested in participating. ST and MM are experienced qualitative researchers and trained interviewers and had no prior relationships with any of the participants.

### Data Collection

While there are numerous modes of data collection when conducting narrative research, our research followed the work of Bertaux and Kohli (1984), who contend that narrative interviews occur in two separate parts (or separate interviews). This approach is consistent with that of other narrative researchers, who recommend that narrative interviews should be in-depth and unstructured (Flick, 1998; Riessman, 1993). In particular, they posit that the first interview often acts as the site of narrative production by which the interviewer asks an open-ended question and probes for more detail as the interview and narration progresses (Bertaux & Kohli, 1984). The first interview began by asking the question: “Can you tell me, in as much detail as you can, what it’s like to be a child life specialist working within an adult oncology center?” The interviewers (ST and MM) probed in response to this question and asked additional open-ended questions for the remainder of the interview.

The second interview is an opportunity to elucidate more details and stories with participants by asking additional questions (sometimes clarifying, sometimes new) based on analysis of their initial interview, as well as the interviews of other participants. The questions asked in the second interview elicited details for which the interviewers sought further clarification and/or were new questions that were developed for the second interview based on the research team’s interpretations of the stories narrated in the first interviews. Additionally, a child life specialist on the research team (HM) provided journal entries that she had written during her experiences working in adult oncology at a tertiary care center to provide context for the development of these interview questions.

All participants provided informed consent prior to the first interview. In total, four child life specialists were recruited to participate in this study. This included some child life specialists from two different institutions. In part, this sample size reflects the small number of individuals doing child life work in adult oncology settings. While many research approaches require high participation rates to achieve power or ensure that their findings are generalizable to wider audiences, narrative scholars place importance within the transferability of findings to others within similar experiences in varied contexts (Riessman, 2008). The richness of the stories collected in narrative research provides a level of depth that does not necessitate the same metrics to satisfy recruitment goals (Connelly & Clandinin, 1990; Lieblich et al., 1998; Tuckett, 2004), and the adequacy of data is determined by participants’ ability to speak at length, and in great detail, about their experiences. Considering this, we recruited a limited number of participants who offered rich stories of their experiences working in adult oncology rather than recruiting a large number of participants with the goal of reaching data saturation. Thus, while only four individuals participated in the study, it is important to note that each took part in two in-depth narrative interviews that ranged between 45 minutes to two and a half hours, establishing a level of rigor consistent with narrative approaches in health research (Mancuso & Polzer, 2010). Due to public health restrictions, interviews were completed over Zoom conference or by phone. The interviews were transcribed verbatim, and a de-identified copy was shared with participants after each interview. As mentioned, one participant provided supplemental journal entries of their experiences working in adult oncology. These journal entries were created prior to the study’s development, serving as a method to document their experiences in a new role. These journal entries were analyzed alongside the transcripts from the interviews, incorporating insights about their experiences into the narrative themes identified. Each participant was also assigned a pseudonym.

### Data Analysis

Narrative analysis can be completed using a variety of different approaches. Some narrative researchers contend that researchers should develop their own analysis methods for their studies and may combine different approaches (Riessman, 2008; Smith & Sparkes, 2008). As such, we used a combination of approaches to analyze the participants’ stories and generate narrative themes (Lieblich et al., 1998; Riessman, 2008). Initially, two study team members (ST and MM) did multiple close reads of the transcripts, while considering how child life specialists conceptualized their experiences in adult oncology. ST and MM documented patterns, thoughts, similarities, and differences they noticed between the participant stories and accounted for their body language, tone of voice, and phrasing of stories. These developed patterns were shared with the wider team (MV, DLL, DB, HM, and JS) for additional input and were further investigated in the second interview. The wider team was presented with the transcripts and developed patterns, to see if they identified similar interpretations. They also provided methodological input by pinpointing areas that could benefit from further exploration in the second interview. The themes were further refined after multiple close reads of the second interview transcripts. After identifying narrative themes within and between interviews, they were interpreted by ST and MM. The interpretations were shared with clinical collaborators (DL, HM, and JS) with previous experience in oncology to see if these interpretations resonated with their knowledge and experiences. The clinical collaborators also had the opportunity to identify any nuances or contexts that were overlooked, as well as any misinterpretations.

#### Rigor and Reflexivity

Several steps were taken to establish rigor during data collection and analysis. Specifically, we followed Tracy’s (2010) criteria, which delineates actions to ensure credibility and sincerity. Credibility is defined as the trustworthiness of the data. This was achieved through analyst triangulation and member reflections (Tracy, 2010). To ensure credibility in the data, we employed analyst triangulation, where both ST and MM conducted the interviews and primary data analysis. This collaborative approach helped to strengthen our findings. To validate and substantiate our interpretations, the findings were shared with the broader team to ensure our interpretations were grounded in the data and aligned with the research question. To further enhance the credibility of our study, during the second interview, participants were provided with an overview of our initial findings from their interview and those of other participants. This process allowed them the opportunity to clarify and provide additional insights and details, contributing to a more comprehensive and robust analysis. Sincerity, which is being honest and transparent about the researcher’s biases and goals, influences how the study is conducted (Tracy, 2010). Researchers can establish sincerity by being transparent about the research process. In order to be transparent, we completed a formal audit trail during the study to document all research activities and decisions. This also ensures that our research is reproducible. We also established sincerity through self-reflexivity, where all research team members reflected on their own personal experiences, motivations, and abilities to conduct this study. At the time of data collection and analysis, ST was a master’s student experienced in qualitative research, learning narrative methodology. The research project was conducted under the supervision of two experts, MM, a qualitative health researcher who focuses on exploring moral distress among healthcare providers in oncology and primary care, and MV, a qualitative health researcher who examines ethical complexities in various clinical settings. Additionally, the team included members with diverse backgrounds and expertise. HM is a child life specialist, that previously worked in adult oncology. DB is a research coordinator in oncology. DLL is a pediatric palliative care specialist and JS is a medical oncologist for adults, both of whom have worked with child life specialists. Additionally, the research team continuously reflected on their experiences and were aware of our positionality through the analysis of stories and how our positionality had a bearing on the interpretations of the data. Research team members were encouraged to make note of any time their interpretations changed or substantiated.

## Results

The four child life specialists participating in this study had a wide range of experiences and involvement in adult oncology. Their experience ranged from less than 1 year to 14 years. Two child life specialist participants (Aesha and Daisy) took on adult oncology cases on an as-needed basis (ranging from 5 to 10 cases), one (Madison) worked full-time in an adult oncology center, and one (Lucy) worked part-time in adult oncology and part-time in a different department as a child life specialist. Narratives about each participant and the main contours of their stories are provided in Table 1.

**Table 1.** Participant Narratives.

Participant	Narrative
Aesha	Aesha depicted her experiences in adult oncology in short stories, often comparing the stories to scenarios in her other child life work in different disciplines. Her stories often focused on end-of-life cases that she consulted on; she narrated these stories at length and often drew attention to the importance of legacy work, a term used to describe activities completed with the family to create a tangible item for the child to cherish after their parent had died. The researchers noticed that, when speaking about these experiences, Aesha often tried to minimize the amount of work she did, frequently reminding the interviewer that she took on only a few cases. Sometimes within these narratives, Aesha mentioned names of child life specialists who may provide better or more comprehensive stories than her. Suggested within these narratives was some fear that she had minimal experience and these consults were not good enough to share.
Daisy	Daisy narrated her stories in long monologues, giving detail to particular cases she felt were rewarding in adult oncology. Daisy emphasized the value of involving child life in adult oncology to bring developmentally important information to not just the children but also the patients. Daisy described in multiple stories that patients have little understanding of their diagnosis due to their health literacy, and the child life specialist role helps clarify misconceptions about their diagnosis. In one of her narrations, Daisy expressed the emotional labor involved in many end-of-life cases she consulted on. She mentioned distancing herself from end-of-life cases due to her own recent and personal experiences with death, making emotionally intense conversations difficult to partake in professionally. She stated that she would only take on these cases to support families if there was nobody else.
Madison	Madison described her experiences working in adult oncology in long monologues, often with ambivalence in highlighting both the challenging and fulfilling aspects of the role. Within her stories, Madison mentioned that this role was her first employed child life position outside of her internships and was one of the hardest but the most rewarding thing she has ever done in her professional life. Her stories often focused on the challenges involved in navigating and supporting children during the pandemic, especially for end-of-life cases. In these scenarios, Madison often had to take time to detach or debrief as these conversations evoked a lot of emotions. Demonstrating a notable ambivalence, she also expressed at length how rewarding it was to provide support to children and be a part of their journey. She often referenced back to creating a family tree (a legacy activity) with kids, as it provided a tangible item that kids and other family members could cherish for the rest of their lives. Madison mentioned that these interviews helped her feel validated in her role and allowed her to share her unique experiences, suggesting that she felt isolated as the only child life specialist in adult oncology at the time.
Lucy	Similar to Daisy and Madison, Lucy also narrated her experiences in long monologues. Her narration began by describing how she transitioned from pediatric oncology into adult oncology due to the increase in referrals for support. Unlike the other participants, Lucy’s stories seemed more focused on the positive aspects of the role, often describing how valued she felt by her team. She only briefly expressed some challenges that were mainly due to institutional constraints. In particular, Lucy mentioned that in some instances when a parent dies, she can sometimes refer the child out to another counselling service for grief and bereavement support. Scenarios like this seemed bittersweet, as the child was continuing to get support, but they no longer get to support the child. Later in the first interview, Lucy stated that she had transitioned into that grief and bereavement support role in an outside agency as she wanted to continue providing support to the children.

The participants’ narratives often highlighted the challenges of working in an adult oncology environment. Among the challenges they faced, two challenges were repeatedly discussed in their stories. The first challenge was described as difficulties balancing the wishes of parents against the perceived needs of the child(ren). This was an equilibrium that regularly occurred, as child life specialists tried to align the needs of both the child and parent, all with the goal of creating the best outcome for the child. In addition to the tensions between parents and children, participants shed light on the systemic limitations that prevented them from providing optimal care to the child(ren). These limitations ranged from late involvement in cases to a lack of bereavement support for the child.

### “It’s a Bit of a Dance”: Tensions Between the Needs of Parents and Their Children

One of the primary goals of CLS interventions is to ensure children receive honest and developmentally appropriate information about the illness with the goal of improving their experience and long-term outcomes. Accordingly, a common thread woven among the participants’ narratives was the work they did tailoring and providing education to families, often about diagnostic and prognostic information. However, their stories made apparent their experiences of tension between the family dynamic and their clinical expertise, including knowledge of child development. Their stories drew attention to how, at times, families may be hesitant and unreceptive to involving child life services for their minor children, even when transparency and honesty about parental diagnoses and prognoses would be beneficial for their children according to the clinical expertise of the child life specialists. Their descriptions of their clinical services emphasized the value that conversations regarding the parent’s diagnostic and prognostic information can have for the child and their psychological well-being. As an example, Madison described a scenario where a parent continued to delay disclosing her initial diagnosis to her son for roughly 11 months, all the while the son knew about the diagnosis yet felt uncomfortable bringing it up with the parent.

The tension in parents wanting to shield their children was evident in a story narrated by Aesha, in which she described the most distressing cases as involving families that were avoiding disclosing the diagnosis with their children even when the parent was at the end of their life: “Maybe someone that’s at end of life and the parents haven’t told the kids anything and the kids don’t know, you know, even though their parent has cancer, and you know the parents don’t want to include the children and they’re trying to protect them” (Aesha). Through the phrasing of “protect,” she draws attention to how parents believed that the best interests of the children were served by not disclosing information regarding their prognosis or imminent death. This, however, conflicted with what the child life specialists believed to be in the best interest of the children.

Daisy went on to highlight that without these conversations about a parent’s condition, the child may experience negative future consequences:

[…] the other pieces when you look at children who’ve been kept in the dark about things, there are trust issues with people. Um, you know, they don’t have the ability to have appropriate closure with the adult that has died. Um, there can be a lot of resentment between them and their parent that is surviving. (Daisy)

Through this narration, Daisy suggests that child life specialists seem to worry about the immense responsibility of fostering future relationships between children and their parent(s). Our participants’ stories conveyed that child life specialists feel they can not only help the children build bonds with their parent(s) and stay informed during their illness but also help the children build trust and coping skills to maintain healthy relationships in the future.

Through the quotes above and other narrations told by the child life specialists, there is an implied gravity to being unable to tell children about their parent’s illness; the child life specialists assumed responsibility not only for the children now but also for their future. Through their stories, our participants asserted that if they do not bridge conversations between parents and their children, not only will the children be “robbed of some time with their parents” (Lucy) while they are living but they may further be “robbed” of coping mechanisms and trusting relationships with their remaining parent in the future.

As participants narrated how their clinical expertise gives them an understanding of development patterns and coping skills, their stories of feeling constrained by the families were interpreted as something that was challenging to navigate. They often were trapped between what they knew and believed to be “right” (telling the children about their parents’ condition) but constrained from doing so by another professional tenet (abiding by the parents’ wishes). This tension was narrated by Madison, who described an instance in which a mother did not want any diagnostic or prognostic information shared with her child. In her story, she explicitly stated that families do not mean any harm to their children in limiting conversations about their prognosis, but changing their perspective and getting their permission, at times, is “a bit of dance”:

[…] it’s always kind of a bit of a dance around that because at the end of the day, it’s---it’s her choice and it’s her decision and that’s her child and that’s what she feels is best, I think, recognizing that most parents, if not all parents, are coming from a really well intended place, but then offering, what we know and what we see of the things that are helpful for kids to be able to cope, and really trying to promote what the benefits of providing kids with open and honest information are and asking families like “can I share a little bit with you about what I’ve seen other families in your situation do, and what they’ve said is helpful and what they've shared from their experiences,” and I think just trying to build trust and rapport with parents and---and making sure that they know you’re just trying to offer as much guidance and support from my area of expertise, as well as you know what I know from the other families that I’ve worked with, but, um that ultimately it is their decision and I’m still there to support them, no matter what they decide. (Madison)

By describing the different benefits of having these conversations, in addition to outlining the different techniques she uses to converse with parents about allowing these conversations to occur, Madison draws attention to the tension she feels in wanting to abide by the parent’s wishes while trying to do right by their children. Additionally, her repeated phrasing of “at the end of the day it’s her choice” and “ultimately it is their decision” suggests that she finds these decisions to be challenging and uses this phrasing as a form of affirmation to herself and the interviewer that she did what she could to support both the parent and the child and that it was not a personal or professional failure to practice in an optimal way.

### “There Is So Much More Support We Could Have Provided”: Barriers to Optimal Care

The child life specialists were all very passionate about providing the best care for children and their families, and their narratives describe how emotionally involved they are when supporting the children through this difficult journey. However, these participants described challenges within the adult hospital system that prevented them from providing the best possible care, including unfamiliarity with the role, late referrals, abrupt ends to relationships, limited options for ongoing support, and the absence of follow-up. These challenges often left them frustrated and exhausted with the system.

The child life specialists noted that the unfamiliarity of their role to the medical team often posed challenges when providing support to families. Madison shared her experience working as a full-time child life specialist in an adult environment, where she was frequently overlooked:

I’ve been here a year and a half and I’m still like left out of important emails that go around, like not included in some meetings that happen, my input isn’t, you know, asked for, for things that are like related directly to me, like my referral process or like you know, those kinds of things. (Madison)

Due to the medical team’s familiarity with the role of child life specialists in adult oncology, our participants frequently narrated that they were called in to assist the families very late, often when the patient was imminently dying. This challenge was often distressing as they were now charged with the responsibility of covering all topics related to a cancer diagnosis (and now death), including diagnosis explanation, change of status, and end of life and legacy—all in one encounter. Our participants drew attention to feeling unable to practice to their full scope and the challenges that this presents. Madison spoke about these situations where she was unable to give the time and resources needed to promote thorough and thoughtful conversations about these varied topics and ensure that the family was doing well:

[…] the patient is at end of life and that’s when the family is agreeable to services and I guess it’s challenging to know that there could have been so much more support provided before that moment, and then that can be a really difficult time to help kids when there’s so much more support we could have provided earlier on […]. (Madison)

In this scenario, Madison’s frustration with the system was evident through her repeated phrasing of “so much more support,” suggesting that she believed she could have done more for the family if she had been brought in earlier to help and if there was a mutual understanding of her role in supporting the child(ren). Her words impart her feelings of guilt and dismay that the family did not get as much support as they could have and the support she offered was extremely limited by time. Madison elaborates that when she is involved in cases at the earlier stages, she can provide small pieces of information to the child(ren) at a time, through engaging in activities. This approach allows them to process the information at their own pace and allows them to ask questions in subsequent appointments, rather than in one encounter when death is imminent.

Similarly, Lucy described a scenario where the children may know about the cancer but not its incurable nature, and how the child life specialists would have to squeeze a disclosure and legacy work into a tight window especially when they were brought in late for urgent cases. They explained that disclosure involves a conversation that the ill parent is terminal and that despite all of the treatments they tried, they are going to die. After disclosing the prognosis, child life specialists facilitate legacy work, which involves creating tangible items such as handprints, a family tree, or photos that the child can keep to remember their dying parent.

Preparation for a disclosure requires the child life specialist to mentally prepare for a “really hard conversation,” arrange art supplies, sometimes plan with the care team (who may or may not be present during the disclosure), and ensure that the physical space is emotionally safe for the children. Already working in a constrained time period to ensure all of this is done for the family and children, these cases were made exponentially more difficult and distressing when cases do not go according to the plan, particularly when the patient died earlier than expected before there was an opportunity to discuss with the children:

And so now I’m like “Okay, you know, maybe I lost sleep over the fact that I’m gonna have to help share with these kids that their dad is dying,” which, no matter how many times I do these conversations I have, um, have gathered, a box of supplies now I have to put all that stuff away, and wipe all that out of my mind and then I can focus on the day. That’s, not to say it easier or harder than actually going and having the conversation, but when it kind of is that abrupt ending, it’s just that, it’s very abrupt. Um, but then, you also want to make sure that the kids are kind of handed off to an agency or an organization or that the family has the tools to be able to help the kids now and there’s no way of knowing if they do or don’t, which can be very hard. (Lucy)

Through Lucy’s narrative, she explicitly states how important these conversations are for her, specifically acknowledging that she loses sleep from being brought in at a very late point for the intervention. In her phrasing of “share with these kids, that you know, their dad is dying,” however, she seemingly is attempting to draw away from her frustration, emphasizing that her feelings don’t matter because at the end of the day these “kids” are losing their dad.

Lucy also elaborated in the above on how the abrupt ending, lack of closure, and uncertainty regarding ongoing support are additional challenges. She expressed guilt in that there was little support or closure for the children in these circumstances. Participants described that children were usually not registered as hospital patients for child life support, as the patient in the system was the ill parent. If the patient died, the relationship between the child and the child life specialist ends with little opportunity for post-mortem guidance or support for grief and bereavement. Aesha described the regret with cases ending abruptly:

I don’t ever know kind of what happens with them, like how things turned out, or like I don’t kind of get the follow-up, like “oh, you know mom died this day and kids are coping well,” um, I find that in that in those urgent referrals, I just kind of give support in the moment, and then I don’t really know what happens, so I think that can be kind of hard just because you don’t kind of have any level of closure or just don’t really know what happened. (Aesha)

The child life specialists expressed these cases are often emotionally challenging as they cannot always ensure that the child will get support from an external source. They do not get to follow up, know what happens, or have a sense of closure, but they still feel obligated to do right by the child in some way. This was expressed by Lucy, who, in her narrative, describes distress stemming from a lack of “child life coverage” and the structure of patient registration in the hospital system. Once again, assuming responsibility for the children’s future, she narrated that there is no way to ensure children are handed off to an external agency for ongoing grief support, setting them up for success later in life:

[…] not having enough of that child life coverage that’s not to say, like you need someone through the night, but what I mean is even just in terms of having more resources, even within the community to say you know I’m going to connect you with you know this external organization, so that way, at least, you know if this person dies overnight, maybe I won’t be able to look after them, but I’m very confident that someone else will, whereas if they go home and they’ve not been given any of those resources or any of that information they’re just kind of left to cope on their own, which happens all the time, but when it was one where like you are prepared to provide the support and you were prepared to intervene, you’re prepared to do all these things, and then suddenly you’re not able to anymore it’s just it’s just it’s tiring [laughs]. (Lucy)

In this narrative, Lucy emphasized how she wants to do the right thing for the child by finding external resources for support, but systemic limitations restrict her from doing so. In her phrasing “which happens all the time,” she draws attention to how this is a consistent challenge, which has become exhausting to deal with, but she has had to accept that there is not anything she can do about these situations.

## Discussion

The purpose of our study was to explore the experiences of child life specialists working in adult oncology. We conducted two interviews with each of the four child life specialists who work with adult cancer patients and their minor children, to elucidate their caregiving stories. These narratives highlight the complexities of working as a child life specialist in an adult environment. The participants suggested that the cases they consulted on were often morally challenging, as they were unable to do what they believed was the right thing for the children. These challenges were often rooted in trying to balance support to the children with the parent or caregiver’s wishes regarding disclosure of medical information to their children, being brought into cases only at end of life, and the lack of follow-up or bereavement support for the children in the hospital. To our knowledge, this is the first study to describe child life specialists’ experiences working in an adult environment. As there is increasing awareness of the value of child life in adult environments, these findings provide insight into some of the challenges they have experienced and the significant amount of support still needed for success in these roles and for families.

The child life specialists in our study highlighted some complexities of family-centered care, particularly when the “patient” is the family. Family members are actively in the care and decision-making of the child, but challenges can arise when the family’s perspective does not align with the child life specialist (Lookabaugh & Ballard, 2018). For instance, they mentioned in their stories that sometimes parents withhold information about their cancer diagnosis or prognosis from their children, intending to protect them from difficult emotions. The participants suggested there was some tension in these situations as some parents are reluctant, but the child life specialists know the value of having these open and honest conversations. This tension is similar to that experienced by pediatric oncology providers who provide family-centered care and have to navigate parents’ wishes of not disclosing prognostic information to children diagnosed with cancer (Rost & Mihailov, 2021; Sisk et al., 2016). Our participants emphasized that withholding diagnostic information could have negative repercussions for the child. For instance, our participants mentioned how withholding information may lead to resentment and trust issues with adults. Previous literature has reported that children may experience anger, guilt, frustration, disappointment, and unresolved grief because they were uninformed and did not have time to say goodbye to their dying family member; these experiences and children’s reaction to the death of a family member are also influenced by other factors (Bylund-Grenklo et al., 2016; Harris, 1991). Our participants suggested that children who are aware of the cancer prognosis may experience similar repercussions if they feel that important information about the details of the diagnosis or prognosis has been withheld. Research has described that children often have questions and want information on their parent’s cancer diagnosis and prognosis but are unsure how to initiate these conversations (Kennedy & Lloyd-Williams, 2009b; Patterson & Rangganadhan, 2010; Wray et al., 2022). They may want an opportunity to speak with a healthcare professional to ask questions about their parent’s cancer diagnosis but are rarely presented with an opportunity to speak frankly and at length with someone who has the necessary clinical and social expertise to facilitate these difficult conversations (Wray et al., 2022). For example, while oncology providers have significant clinical expertise, they may lack confidence in their ability to facilitate disclosure conversations with minor children (Sisk et al., 2016). Child life specialists could help fill this gap, translating medical content to children in a way they can understand and absorb this information. Through this work, child life specialists could be an act of support person for the child. They can provide open and honest information about the cancer prognosis while helping the child develop coping skills for ongoing and future relationships.

Barriers to providing optimal care were described by the participants, such as being brought in last minute and the lack of continuous care post-mortem. For instance, child life specialists’ stories highlighted how end-of-life cases were as emotionally challenging since they were brought in for support very late. Participants expressed it was often difficult to build rapport with the child and family in a tight window of time while cramming in information about diagnosis and prognosis. In other cases, the relationship with the child would end abruptly and they would not know if the child was coping with the death of a loved one or receiving support. For child life specialists, continuity of care is an important aspect of their services, and they have much to contribute to post-mortem healing and development. This experience might be unique to child life specialists working in adult oncology in a hospital environment. For instance, child life specialists in adult community settings such as hospice and palliative care may have additional time and resources to build rapport with the family and address all the child’s needs (Hernandez et al., 2023). Hospice and palliative care community settings also offer grief and bereavement programs, allowing the child life specialist to develop longitudinal relationships with the child and family, as well as continue providing support after the family member has died. This way, the child can receive support from an adult they already trust and partake in forms of support such as peer support groups, weekend treatments, and summer camps (Hernandez et al., 2023). The child life specialist can continue providing customized care and feel at ease knowing the child is still receiving some type of psychosocial support after losing their loved one. Since hospice workers are repeatedly exposed to traumatic events (e.g., death), the child life specialists in these settings have additional supports to help cope with any emotional distress when working with children (Hernandez et al., 2023).

### Implications for Policy, Clinical Practice, and Research

The findings from our study highlight the importance of institutional support in order to provide the highest quality of care to child life specialists’ clients. This support includes facilitating institutional awareness of the issues and needs of oncology patients with minor children and those children. Recognizing the importance of the CLS role in the healthcare team and adult environments is crucial, as it will empower them to carry out their interventions effectively. Additionally, understanding the value and necessity of child life for family outcomes and providing sufficient resourcing such as staffing, funding, and materials to meet demands are important. Acknowledging and understanding the value of child life can also allow for the development of spaces for child life specialists to speak about their experiences. Creating collaborative external partnerships with advocacy and community locations can better support patients and their families, and the continuum of care. The addition of these institutional resources and support could lead to standardized integration of child life specialists at the time of diagnosis and continuing grief and bereavement support after a death. Future research should investigate the experiences of child life specialists working in other adult environments, such as critical care or community settings to determine if additional changes are needed to better support child life specialists in these roles.

### Strengths and Limitations

To our knowledge, this is one of the first studies to explore child life specialists’ experiences in adult oncology. Our interpretations described an original health human resource model that fills a gap in an important clinical area. This model involves the integration of child life specialists in an adult environment to meet the needs of cancer patients with minor children, as well as the needs of those children themselves. The use of narrative inquiry allowed us to gather rich stories of how child life specialists navigate their roles and experiences within a new institutional context, providing us with detail and context previously unexplored. Our sample of four child life specialist respondents is small but adequate for the in-depth narrative study we conducted. Our data included very few stories of times when child life specialists were involved early in a case. This is likely because the child life specialists that were interviewed were working in a new institution, and as a result, their stories focused on the lack of awareness of their roles and the challenges with it. In an environment where child life specialists are more integrated and established, the opportunity for early involvement may yield different experiences for the family and the clinical team, but our data is unable to comment on this.

## Conclusion

Child life specialists working in an adult oncology setting highlighted several challenges in their current roles, including wanting to be honest with the child while respecting the patient or caregiver’s wishes. Other times, these challenges involved barriers to providing care earlier or continuing grief and bereavement care after the death of the adult patient. There is an opportunity for institutions to provide additional support for the children of adult oncology patients by employing and supporting child life specialists working in these environments.

## Data Availability

The data given in this article are datasets generated for this study and are not publicly available because participants did not consent to the use of their interview data beyond the research team, due to the potentially identifying nature of entire transcripts. Readers are welcome to contact Dr. Monica Molinaro for further information.

## Appendix

### Abbreviation

CLS: Child life specialist

## References

[bibr1-10497323231215950] Alexander E. O’Connor M. Rees C. Halkett G. (2019). A systematic review of the current interventions available to support children living with parental cancer. Patient Education and Counseling, 102(10), 1812–1821. 10.1016/j.pec.2019.05.001 31109770

[bibr36-10497323231215950] Association of Child Life Professions (2022). What is a certified child life specialist? https://www.childlife.org/the-child-life-profession

[bibr2-10497323231215950] Atli Özbaş A. Kovanci M. S. Köken A. H. (2021). Moral distress in oncology nurses: A qualitative study. European Journal of Oncology Nursing, 54, 102038. 10.1016/j.ejon.2021.102038 34601227

[bibr3-10497323231215950] Bertaux D. Kohli M. (1984). The life story approach: A continental view. Annual Review of Sociology, 10(1), 215–237. 10.1146/annurev.so.10.080184.001243

[bibr4-10497323231215950] Bruce J. E. McCue K. (2018). Child life in the adult ICU: Including the youngest members of the family. In Families in the intensive care unit (pp. 365–379). Springer.

[bibr5-10497323231215950] Bylund-Grenklo T. Fürst C. J. Nyberg T. Steineck G. Kreicbergs U. (2016). Unresolved grief and its consequences. A nationwide follow-up of teenage loss of a parent to cancer 6–9 years earlier. Supportive Care in Cancer, 24(7), 3095–3103. 10.1007/s00520-016-3118-1 26899858

[bibr6-10497323231215950] Connelly F. M. Clandinin D. J. (1990). Stories of experience and narrative inquiry. Educational Researcher, 19(5), 2–14. 10.3102/0013189x019005002

[bibr7-10497323231215950] Crotty M. J. (1998). The foundations of social research: Meaning and perspective in the research process. In The foundations of social research, (pp. 1–256). Sage Publications.

[bibr8-10497323231215950] Davey M. P. Niño A. Kissil K. Ingram M. (2012). African American parents’ experiences navigating breast cancer while caring for their children. Qualitative Health Research, 22(9), 1260–1270. 10.1177/1049732312449211 22767699

[bibr9-10497323231215950] Ellis S. J. Wakefield C. E. Antill G. Burns M. Patterson P. (2017). Supporting children facing a parent's cancer diagnosis: A systematic review of children's psychosocial needs and existing interventions. European Journal of Cancer Care, 26(1), e12432. 10.1111/ecc.12432 26776913

[bibr10-10497323231215950] Emden C. (1998). Conducting a narrative analysis. Collegian, 5(3), 34–39. 10.1016/s1322-7696(08)60299-1 9887715

[bibr11-10497323231215950] Fisackerly B. L. Sira N. Desai P. P. McCammon S. (2016). An examination of compassion fatigue risk in certified child life specialists. Children's Health Care, 45(4), 359–375. 10.1080/02739615.2015.1038716

[bibr12-10497323231215950] Flick U. (1998). An introduction to qualitative research. In An introduction to qualitative research (pp. 1–100). Sage Publications.

[bibr13-10497323231215950] Guan T. Nelson K. Otis-Green S. Rayton M. Schapmire T. Wiener L. Zebrack B. (2021). Moral distress among oncology social workers. JCO Oncology Practice, 17(7), e947–e957. 10.1200/OP.21.00276 34252313 PMC8462654

[bibr14-10497323231215950] Harris E. S. (1991). Adolescent bereavement following the death of a parent: An exploratory study. Child Psychiatry and Human Development, 21(4), 267–281. 10.1007/BF00705931 1855398

[bibr15-10497323231215950] Hernandez D. E. Tresnan E. Taylor-Brickey M. (2023). Palliative care and hospice. In The role of child life specialists in community settings (pp. 63–88). IGI Global.

[bibr16-10497323231215950] Huizinga G. A. Visser A. Zelders-Steyn Y. E. Teule J. A. Reijneveld S. A. Roodbol P. F. (2011). Psychological impact of having a parent with cancer. European Journal of Cancer, 47(Suppl 3), S239–S246. 10.1016/S0959-8049(11)70170-8 21943981

[bibr17-10497323231215950] Humphreys C. LeBlanc C. K. (2016). Promoting resilience in paediatric health care: The role of the child life specialist. In DeMichelis C. Ferrari M. (Eds.), Child and adolescent resilience within medical contexts: Integrating research and practice (pp. 153–173). Springer International Publishing. 10.1007/978-3-319-32223-0_9

[bibr18-10497323231215950] Inhestern L. Bultmann J. C. Johannsen L. M. Beierlein V. Möller B. Romer G. Koch U. Bergelt C. (2021). Estimates of prevalence rates of cancer patients with children and well-being in affected children: A systematic review on population-based findings. Frontiers in Psychiatry, 12, 765314. 10.3389/fpsyt.2021.765314 34899425 PMC8656299

[bibr19-10497323231215950] Kennedy V. L. Lloyd-Williams M. (2009a). How children cope when a parent has advanced cancer. Psycho-Oncology, 18(8), 886–892. 10.1002/pon.1455 19137509

[bibr20-10497323231215950] Kennedy V. L. Lloyd-Williams M. (2009b). Information and communication when a parent has advanced cancer. Journal of Affective Disorders, 114(1–3), 149–155. 10.1016/j.jad.2008.06.022 18684513

[bibr21-10497323231215950] Laor-Maayany R. Goldzweig G. Hasson-Ohayon I. Bar-Sela G. Engler-Gross A. Braun M. (2020). Compassion fatigue among oncologists: The role of grief, sense of failure, and exposure to suffering and death. Supportive Care in Cancer, 28(4), 2025–2031. 10.1007/s00520-019-05009-3 31392551 PMC7223813

[bibr22-10497323231215950] Levesque J. V. Maybery D. (2012). Parental cancer: Catalyst for positive growth and change. Qualitative Health Research, 22(3), 397–408. 10.1177/1049732311421617 21890711

[bibr23-10497323231215950] Lieblich A. Tuval-Mashiach R. Zilber T. (1998) Narrative research: Reading, analysis, and interpretation (Vol. 47). Sage.

[bibr24-10497323231215950] Lookabaugh S. Ballard S. M. (2018). The scope and future direction of child life. Journal of Child and Family Studies, 27(6), 1721–1731. 10.1007/s10826-018-1031-6

[bibr25-10497323231215950] Lysecki D. Bainbridge D. Akitt T. Georgiou G. Meyer R. M. Sussman J. (2021a). Feasibility of a child life specialist program for oncology patients with minor children at home: Demand and implementation. Journal of Clinical Oncology, 39(28 suppl), 28. 10.1200/JCO.2020.39.28_suppl.28

[bibr26-10497323231215950] Lysecki D. Bainbridge D. Akitt T. Georgiou G. Meyer R. M. Sussman J. (2021b). Feasibility of a child life specialist program for oncology patients with minor children at home: Qualitative analysis. Journal of Clinical Oncology, 39(28 suppl), 30. 10.1200/JCO.2020.39.28_suppl.3032822275

[bibr27-10497323231215950] Mancuso F. Polzer J. (2010). “It's your body but...”: Young women's narratives of declining human papillomavirus (HPV) vaccination. Canadian Woman Studies.

[bibr28-10497323231215950] McGee K. (2003). The role of a child life specialist in a pediatric radiology department. Pediatric Radiology, 33(7), 467–474. 10.1007/s00247-003-0900-2 12819835

[bibr29-10497323231215950] Morris J. Turnbull D. Preen D. Zajac I. Martini A. (2018). The psychological, social, and behavioural impact of a parent's cancer on adolescent and young adult offspring aged 10–24 at time of diagnosis: A systematic review. Journal of Adolescence, 65, 61–71. 10.1016/j.adolescence.2018.03.001 29549783

[bibr30-10497323231215950] Morris J. N. Martini A. Preen D. (2016). The well-being of children impacted by a parent with cancer: An integrative review. Supportive Care in Cancer, 24(7), 3235–3251. 10.1007/s00520-016-3214-2 27079580

[bibr31-10497323231215950] Munn E. K. Berber C. E. Fritz J. J. (1996). Factors affecting the professional well-being of child life specialists. Children's Health Care, 25(2), 71–91. 10.1207/s15326888chc2502_1

[bibr32-10497323231215950] Niemelä M. Väisänen L. Marshall C. Hakko H. Räsänen S. (2010). The experiences of mental health professionals using structured family-centered interventions to support children of cancer patients. Cancer Nursing, 33(6), E18–E27. 10.1097/NCC.0b013e3181ddfcb5 20555258

[bibr33-10497323231215950] Patterson P. Rangganadhan A. (2010). Losing a parent to cancer: A preliminary investigation into the needs of adolescents and young adults. Palliative and Supportive Care, 8(3), 255–265. 10.1017/S1478951510000052 20875169

[bibr34-10497323231215950] Phillips F. Prezio E. A. (2017). Wonders and worries: Evaluation of a child centered psychosocial intervention for families who have a parent/primary caregiver with cancer. Psycho-Oncology, 26(7), 1006–1012. 10.1002/pon.4120 26954773

[bibr35-10497323231215950] Piazza J. Brescia A. Heering L. Jenkins J. Peters S. Dwyer-White M. Deeb G. M. (2021). Crossing the chasm to adult care settings: A journey of discovery and research. https://mydigitalpublication.com/publication/?m=62133&i=725784&view=articleBrowser&article_id=4140197&ver=html5

[bibr37-10497323231215950] Rashi C. Wittman T. Tsimicalis A. Loiselle C. G. (2015). Balancing illness and parental demands: Coping with cancer while raising minor children. Oncology Nursing Forum, 42(4), 337–344. 10.1188/15.Onf.337-344 26148313

[bibr38-10497323231215950] Riessman C. (1993). Doing narrative analysis. Narrative Analysis. Sage Publications.

[bibr39-10497323231215950] Riessman C. K. (2008). Narrative methods for the human sciences. Sage Publications.

[bibr40-10497323231215950] Rost M. Mihailov E. (2021). In the name of the family? Against parents’ refusal to disclose prognostic information to children. Medicine, Healthcare and Philosophy, 24(3), 421–432. 10.1007/s11019-021-10017-4 PMC8349339 33847853

[bibr41-10497323231215950] Semple C. J. McCaughan E. (2013). Family life when a parent is diagnosed with cancer: Impact of a psychosocial intervention for young children. European Journal of Cancer Care, 22(2), 219–231. 10.1111/ecc.12018 23231498

[bibr42-10497323231215950] Shields L. Pratt J. Davis L. Hunter J. (2007). Family-centred care for children in hospital. Cochrane Database of Systematic Reviews, (1), CD004811. 10.1002/14651858.CD004811.pub2 17253525

[bibr43-10497323231215950] Shuck A. L. Shuck B. Reio T. G. (2013). Emotional labor and performance in the field of child life: Initial model exploration and implications for practice. Children's Health Care, 42(2), 168–190. 10.1080/02739615.2013.766116

[bibr44-10497323231215950] Simon C. E. Pryce J. G. Roff L. L. Klemmack D. (2005). Secondary traumatic stress and oncology social work: Protecting compassion from fatigue and compromising the worker's worldview. Journal of Psychosocial Oncology, 23(4), 1–14. 10.1300/j077v23n04_01 16618685

[bibr45-10497323231215950] Sisk B. A. Bluebond-Langner M. Wiener L. Mack J. Wolfe J. (2016). Prognostic disclosures to children: A historical perspective. Pediatrics, 138(3), e20161278. 10.1542/peds.2016-1278 27561728 PMC5005028

[bibr46-10497323231215950] Smith B. Sparkes A. C. (2008). Narrative and its potential contribution to disability studies. Disability and Society, 23(1), 17–28. 10.1080/09687590701725542

[bibr47-10497323231215950] Sutter C. Reid T. (2012). How do we talk to the children? Child life consultation to support the children of seriously ill adult inpatients. Journal of Palliative Medicine, 15(12), 1362–1368. 10.1089/jpm.2012.0019 22978620

[bibr48-10497323231215950] Tafjord T. (2021). Managing strong emotions: Nurses' recognition and responses to personal emotions when approaching parents with cancer and their dependent children. Qualitative Health Research, 31(5), 926–941. 10.1177/1049732320983788 33554765

[bibr49-10497323231215950] Tracy S. J. (2010). Qualitative quality: Eight “big-tent” criteria for excellent qualitative research. Qualitative Inquiry, 16(10), 837–851. 10.1177/1077800410383121

[bibr50-10497323231215950] Tuckett A. G. (2004). Qualitative research sampling: The very real complexities. Nurse Researcher, 12(1), 47–61. 10.7748/nr2004.07.12.1.47.c5930 15493214

[bibr51-10497323231215950] Weaver K. E. Rowland J. H. Alfano C. M. McNeel T. S. (2010). Parental cancer and the family: A population-based estimate of the number of US cancer survivors residing with their minor children. Cancer, 116(18), 4395–4401. 10.1002/cncr.25368 20586037 PMC3164357

[bibr52-10497323231215950] Wray A. Seymour J. Greenley S. Boland J. W. (2022). Parental terminal cancer and dependent children: A systematic review. BMJ Supportive and Palliative Care. 10.1136/bmjspcare-2021-003094 35091436

